# Effects of angiotensin II on the cerebral circulation: role of oxidative stress

**DOI:** 10.3389/fphys.2012.00484

**Published:** 2013-01-03

**Authors:** T. Michael De Silva, Frank M. Faraci

**Affiliations:** ^1^Department of Internal Medicine, Cardiovascular Center, The University of Iowa Carver College of MedicineIowa City, IA, USA; ^2^Department of Pharmacology, Cardiovascular Center, The University of Iowa Carver College of MedicineIowa City, IA, USA

**Keywords:** cerebral blood flow, endothelium, cerebral arteries, NADPH oxidase, nitric oxide, neurovascular coupling

## Abstract

Oxidative stress has emerged as a key component of many diseases that affect the vasculature. Oxidative stress is characterized as a cellular environment where the generation of oxidant molecules overwhelms endogenous anti-oxidant defense mechanisms. NADPH oxidases are a family of enzymes whose primary purpose is generation of reactive oxygen species (oxidant molecules) and therefore are likely to be key contributors to oxidative stress. Hypertension is associated with oxidative stress in the vasculature and is a major risk factor for stroke and cognitive abnormalities. Angiotensin II (Ang II) is the main effector peptide of the renin-angiotensin system (RAS) and plays a critical role in promoting oxidative stress in the vasculature. In the cerebral circulation, Ang II has been implicated in reactive oxygen species generation, alterations to vasomotor function, impaired neurovascular coupling, inflammation, and vascular remodeling. Furthermore, studies in humans have shown that cerebral blood flow is altered during hypertension and therapeutically targeting the RAS improves cerebral blood flow. Importantly, many of the aforementioned effects have been shown to be dependent on NADPH oxidases. Thus, Ang II, NADPH oxidases and oxidative stress are likely to play key roles in the pathogenesis of hypertension and associated cerebrovascular disease. This review will focus on our current understanding of the contribution of Ang II and NADPH oxidases to oxidative stress in the cerebral circulation.

## Introduction

Hypertension is a highly prevalent disease throughout the world. For example, it is currently estimated that 1 in 3 adults in the United States has high blood pressure, which is defined as having systolic blood pressure ≥140 mmHg and/or diastolic blood pressure ≥90 mmHg, or being on medication to control their blood pressure (Roger et al., 2011). Hypertension has profound effects on the cerebral circulation and the brain and is a major risk factor for both ischemic and hemorrhagic stroke as well as cognitive abnormalities (Kivipelto et al., 2001; Lewington et al., 2002; O'Donnell et al., 2010). Angiotensin II (Ang II) is a pleiotropic peptide that has multiple effects on the cerebral vasculature. Importantly, Ang II plays a critical role in the pathogenesis of hypertension. Two of the most effective anti-hypertensive therapies, angiotensin converting enzyme (ACE) inhibitors and angiotensin type 1 (AT1) receptor antagonists, target key elements of the renin-angiotensin system (RAS). It has also been established that hypertension causes oxidative stress within the vasculature and Ang II is a key contributor to this effect. Within the cerebral circulation, Ang II stimulates the generation of reactive oxygen species (ROS) and has effects on regulation of vascular tone as well as promoting inflammation and changes to vascular structure. All of these effects can influence cerebral blood flow. Either alone or in combination with other mechanisms, Ang II may underline many of the deleterious effects of hypertension on the cerebral vasculature and brain.

In the present review, we will consider recent evidence implicating Ang II as a cause of oxidative stress specifically in the cerebral circulation. As changes in cerebrovascular function may ultimately impact cerebral blood flow, studies examining the effects of hypertension on cerebral blood flow and the role of Ang II and oxidative stress will also be discussed.

## What is oxidative stress?

**Figure 1:**
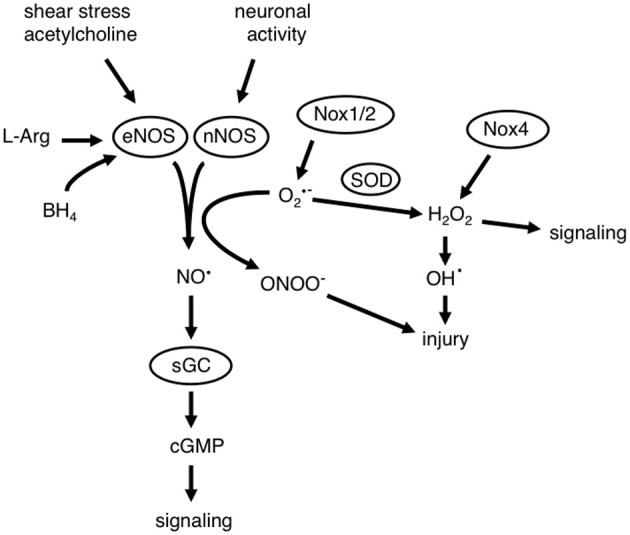
**Interactions between nitric oxide (NO^·^) and reactive oxygen species (ROS).** NO^·^ is a critical component of mechanisms that regulate cerebrovascular homeostasis. Diverse stimuli (including shear stress, neurotransmitters like acetylcholine and activation of neurons) can result in the generation of NO^·^ by either endothelial or neuronal nitric oxide synthase (eNOS and nNOS, respectively). The generation of NO^·^ is dependent on the presence of the substrate L-arginine (L-Arg) and enzyme co-factors including tetrahydrobiopterin (BH_4_). NO^·^ activates its receptor in vascular muscle soluble guanylate cyclase (sGC), which results in the formation of cyclic guanosine monophosphate (cGMP). This results in numerous signaling events and functional effects which include cerebral vasodilation. NADPH oxidases (Nox1 and Nox2) generate superoxide (O_2_^·−^) and Nox4 can generate hydrogen peroxide (H_2_O_2_), ROS can then participate in signaling events, but may also cause cellular injury. O_2_^·−^ is a potent scavenger of NO^·^, which reduces the bioavailability of NO^·^ and results in the formation of peroxynitrite (ONOO^−^), which also causes cellular injury. H_2_O_2_ can react with heavy metals to form the highly reactive and toxic hydroxyl radical (OH^·^).

Oxidative stress is characterized by a shift from a cellular environment where the production and metabolism of oxidant molecules are tightly controlled to one of elevated levels of the same molecules. The excess oxidant molecules may then overwhelm anti-oxidant defense systems, resulting in oxidative stress. Such a shift in balance can occur due to an overproduction of ROS, such as superoxide (O_2_^·−^), or a reduction in the removal of ROS by oxidant defense mechanisms. Key anti-oxidants include O_2_^·−^ dismutases (SOD), glutathione peroxidases, and catalase. ROS can be generated by multiple enzymes within the vasculature as well as non-enzymatic sources. O_2_^·−^ anion is the parent ROS molecule produced by the one electron reduction of molecular oxygen by various oxidases (e.g., NADPH oxidase, cyclooxygenase, enzymes in the mitochondrial electron transport chain, lipoxygenases, cytochrome P450 enzymes). O_2_^·−^ can then be dismutated by SOD, resulting in the generation of hydrogen peroxide (H_2_O_2_; Figure 1). These ROS can undergo further enzymatic or non-enzymatic reactions to generate other powerful oxidant molecules, such as hydroxyl radical (OH^·^), peroxynitrite (ONOO^−^), and hyperchlorous acid (HOCl) (Chrissobolis and Faraci, 2008; Miller et al., 2010) (Figure 1).

Oxidative stress has been implicated in the pathogenesis of many diseases that affect the cerebral circulation and the brain. It is a fundamental component of aging as well as many diseases including hypertension, hypercholesterolemia, atherosclerosis, diabetes, stroke, and Alzheimer's disease. One of the key consequences of oxidative stress is impaired signaling of the vasoprotective molecule nitric oxide (NO^·^), resulting in alterations in vascular function and structure in addition to other effects (Figure 1). Furthermore, oxidative stress contributes to inflammation as well as damage to proteins, DNA and other macromolecules. Thus, oxidative stress has wide implications for overall cerebrovascular function and regulation. As such, it is no surprise that there has been substantial effort devoted to understanding mechanisms that contribute to oxidative stress as well as therapies to reduce oxidative damage.

Many lines of evidence strongly indicate that oxidative stress has damaging effects on vascular function. However, production of ROS *per se* is not always detrimental as ROS sometimes have beneficial effects within the vasculature. Specifically, some ROS can function as vasodilators in cerebral arteries during physiological conditions (Didion and Faraci, 2002; Park et al., 2004; Miller et al., 2005, 2007a; Modrick et al., 2009) as well as during disease (Paravicini et al., 2004; Kitayama et al., 2007). So, while elevated levels of ROS contribute to oxidative stress including vascular damage and dysfunction, lower levels of ROS may be important signaling molecules for some vasoactive stimuli. Cerebral arteries have the capacity to generate significantly higher levels of ROS compared with systemic arteries (Miller et al., 2005, 2009). So, while ROS may serve as an important signaling molecules and vasodilators under physiological conditions, higher levels of ROS may underlie the pathogenesis of disease in the cerebral circulation. Therefore, to presumably avoid potentially harmful levels of ROS, the production and metabolism of ROS are typically tightly controlled.

The presence of oxidative stress has major implications for cerebrovascular function. Oxidative stress has been implicated in impaired cellular signaling, vascular remodeling, and inflammation. Perhaps the best characterized mechanisms by which oxidative stress can impair vascular function is via the disruption of endothelium-dependent NO^·^ signaling. This mechanism involves the chemical reaction between O_2_^·−^ and NO^·^, one of the fastest known biological reactions (Thomson et al., 1995), approximately three times faster than the dismutation of O_2_^·−^ by SOD. Importantly, this reaction not only reduces the bioavailability of NO^·^, but also simultaneously generates the highly toxic reactive nitrogen species (RNS) ONOO^−^, which can have a number of damaging cellular effects (Figure 1). Thus, if levels are increased locally, O_2_^·−^ will rapidly react with and reduce the bioavailability of NO^·^, resulting in oxidative stress. As NO^·^ is a key mediator of cerebral vasodilation driven by both endothelium and neurons, alterations that reduce the bioavailability of NO^·^ would impair these responses (Figure 1).

## What are the sources of ROS that contribute to oxidative stress?

A number of enzymes within the vasculature can be sources of ROS, including xanthine oxidase (Kinugawa et al., 2005), cyclooxygenases (Didion et al., 2001; Niwa et al., 2001), uncoupled NOS (Vasquez-Vivar et al., 1997, 1998; Landmesser et al., 2003; Dikalova et al., 2010; Santhanam et al., 2012), and the mitochondrial electron transport chain (Narayanan et al., 2010). Several of these enzymes generate ROS as either a by-product of normal enzyme activity or when the protein is in a dysfunctional state. By contrast, NADPH oxidases are the only known family of enzymes whose primary function is the generation of ROS (Drummond et al., 2011). Thus, NADPH oxidases are candidates for deliberate ROS production required for normal signaling and for the excessive ROS associated with oxidative stress. It has also been suggested that NADPH oxidase may serve as a source of ROS that initiates a cascade of events including feed-forward mechanisms and recruitment of other enzymes that promote further oxidative stress (Faraci, 2006; Selemidis et al., 2008).

At present study, seven isoforms of NADPH oxidase have been identified. These enzymes are named Nox1, Nox2, Nox3, Nox4, and Nox5 containing NADPH oxidases and DUOX1 and DUOX2. In general, all NADPH oxidases generate O_2_^·−^ by transferring electrons from the substrate, NADPH, via the Nox catalytic subunit to molecular oxygen. Nox1, Nox2, Nox4, and Nox5 containing isoforms are all expressed within vascular cells, however, to date only the Nox1, Nox2, and Nox4 containing isoforms have been identified in the cerebral vasculature (Ago et al., 2005; Miller et al., 2007a). Nox5 containing NADPH oxidase is not expressed in rats and mice and as such, very little is known about this isoform and nothing in regards to any potential role in cerebral arteries. Further studies, potentially including studies of genetic models expressing Nox5 in vascular cells are needed to determine any potential contribution of this isoform to oxidative stress in blood vessels supplying brain. Similarly, our understanding of the expression and contribution, if any, of DUOX1/2 to oxidative stress and vascular disease is extremely limited.

Many studies have implicated a role for NADPH oxidase-derived ROS in models of cerebrovascular disease. As such, the contribution of NADPH oxidases to Ang II-induced oxidative stress will be the focus of this review. However, it is important to remember that other oxidase enzymes and mechanisms may also contribute to oxidative stress during disease.

## NADPH oxidases

Here, we will briefly discuss some of the key features of the three isoforms of NADPH oxidase that have been described within the cerebral vasculature. The detailed structural and regulatory differences between these enzymes have been discussed previously and will not be discussed again here (Bedard and Krause, 2007; Selemidis et al., 2008).

### Structure of Nox1, Nox2, and Nox4 NADPH oxidase

The NADPH oxidase family of enzymes was discovered and initially studied in neutrophils, where Nox2 NADPH oxidase plays a role in immunological host defense. Subsequently, Nox1, Nox2, and Nox4 NADPH oxidases were identified in vascular cells where they are thought to play important roles under both physiological and pathophysiological conditions (Miller et al., 2006).

Nox2 NADPH oxidase consists of two membrane-bound subunits (Nox2 and p22phox), up to three cytosolic subunits [p47phox (organizer subunit), p67phox (activator subunit) and potentially p40phox] and the small G-protein Rac. The catalytic domain resides in Nox2, which contains all of the necessary components to facilitate the transfer of electrons from the substrate, NADPH to molecular oxygen. Specifically, the catalytic domain of Nox2 possesses two haem containing groups and a FAD group, as well as a binding site for NADPH (Taylor et al., 1993; Cheng et al., 2001). The p22phox subunit forms a heterodimer with Nox2 and contains a proline rich region that facilitates the binding of p47phox with the membrane bound subunits (Groemping et al., 2003). As such, p22phox plays a crucial role in regulating Nox2 activity (Ambasta et al., 2004). In unstimulated cells, Nox2 is dormant, and its activation requires the interaction of Nox2/p22phox with the regulatory cytosolic complex containing the p47phox and p67phox subunits. During unstimulated conditions, p47phox is not associated with the Nox2/p22phox complex due to the presence of an autoinhibitory domain which prevents the interaction of p47phox with the proline rich region of p22phox (Groemping et al., 2003). Translocation of p47phox to the membrane bound subunits is dependent on protein kinase C (PKC)-dependent phosphorylation of serine residues on p47phox (Fontayne et al., 2002), which results in a conformational change and displacement of the autoinhibitory region on p47phox (Groemping et al., 2003). p67phox binds to p47phox and this complex translocates to the membrane where two SH3 domains on p47phox bind to the proline rich region of p22phox (Groemping et al., 2003; Nobuhisa et al., 2006). In addition, p47phox has also been shown to bind multiple sites on Nox2, which is believed to be crucial for the final assembly of the active oxidase (Deleo et al., 1995). It has been suggested that the binding of Rac induces a conformation change in p67phox, which in turn promotes its binding to Nox2 (Sarfstein et al., 2004), which leads to a conformation change and electron flow through the Nox2 subunit (Nisimoto et al., 1999). Much less is known regarding the role of p40phox in the activation of Nox2 NADPH. However, in coronary endothelial cells isolated from p47phox-deficient mice, p40phox may act as an alternative organizer subunit in the absence of p47phox (Fan et al., 2009). Currently, it remains to be determined if p40phox is necessary for Nox2 NADPH oxidase activation in the cerebral circulation. The latter point notwithstanding, once the Nox2 NADPH oxidase enzyme complex is assembled, electrons can then be transferred from NADPH to FAD and then to the haem groups and finally to molecular oxygen, thus generating O_2_^·−^.

Nox1 NADPH oxidase is structurally similar to Nox2 NADPH oxidase, but appears to utilize different regulatory cytosolic subunits for its activation. Like the Nox2 subunit, the Nox1 subunit contains two haem groups, FAD and NADPH binding sites, and forms a heterodimer with p22phox in the cell membrane (Ambasta et al., 2004; Kawahara et al., 2005). Nox1 NADPH oxidase is activated by NoxO1 (Nox organizer 1) and NoxA1 (Nox activator 1), which are homologs of p47phox and p67phox, respectively (Banfi et al., 2003). While there is evidence that Nox1 NADPH may be activated by p47phox and p67phox (Banfi et al., 2003), the interaction of Nox1 with NoxO1/NoxA1 is more effective than its interaction with p46phox/p67phox (Banfi et al., 2003). It was recently shown that preventing Rac activation by inhibiting PI3-kinase only attenuates Nox1 NADPH oxidase activity by approximately 20% (Choi et al., 2008), suggesting that it may not be critical for Nox1 NADPH oxidase activity. Unlike p47phox, NoxO1 lacks an autoinhibitory domain and it has been hypothesized that the Nox1 NADPH oxidase might be basally active (Banfi et al., 2003). However, studies using Nox1 deficient mice suggest that Nox1 NADPH oxidase does not contribute to O_2_^·−^ production under basal conditions in cerebral arteries (Jackman et al., 2009).

Nox4 NADPH oxidase was discovered in the kidney (originally named Renox) where it was proposed to serve as an oxygen sensing enzyme (Geiszt et al., 2000). Similar to Nox1 and Nox2 NADPH oxidase, the Nox4 catalytic domain contains all of the required components for oxidase activity and forms a heterodimer with p22phox (Ambasta et al., 2004; Martyn et al., 2006). However, one unique feature of Nox4 NADPH oxidase is that it may not require cytosolic subunits or Rac for oxidase activity and may be basally active (Martyn et al., 2006). Also, Nox4 NADPH oxidase may preferentially generate H_2_O_2_, rather than O_2_^·−^ (Martyn et al., 2006; Schröder et al., 2012) (Figure 1).

### Expression of cerebrovascular NADPH oxidase

Our understanding of the expression profile of NADPH oxidase isoforms in cerebral arteries lags behind that of systemic arteries. However, it has been revealed that mRNA for the Nox1, Nox2, and Nox4 catalytic subunits, p22phox as well as the cytosolic subunits p47phox, p67phox, NoxO1, and NoxA1, are expressed in the rat basilar artery (Paravicini et al., 2004; Ago et al., 2005). Furthermore, Nox1, Nox2, and Nox4 proteins are expressed in cerebral arteries (Miller et al., 2007a). The specific cellular localization of each Nox isoform is distinct. In the vasculature in general, it is believed that Nox1, Nox2, and Nox4 are expressed in endothelial cells, Nox1 and Nox4 are expressed in smooth muscle cells and Nox2 and Nox4 expressed in adventitia (Drummond et al., 2011). Indeed, using immunostaining, Nox1 was shown to be present in all layers of the basilar artery (Ago et al., 2005). Furthermore, using immunostaining and electron microscopy, it has also been demonstrated that Nox2 protein is primarily expressed in endothelial and adventitial cells of cerebral arteries (Kazama et al., 2004; De Silva et al., 2009; Miller et al., 2009). Nox4 immunostaining has been shown to be present throughout the vascular wall (Kleinschnitz et al., 2010).

### Subcellular localization of NADPH oxidases in cerebral arteries

Very few studies have investigated the specific subcellular localization of the different Nox isoforms in cerebral arteries. In rats and mice, electron microscopy has been used to localize Nox2 to the plasma membrane of endothelial cells (Kazama et al., 2004). Furthermore, Nox2 immunoreactivity was also detected in the cytoplasm of endothelial cells (Kazama et al., 2004), although the exact subcellular structure where Nox2 was located was not determined. While we currently lack further detail regarding the intracellular localization of Nox isoforms in cerebral vessels, work in cultured vascular cells has revealed differences in the localization of the Nox isoforms. In cultured endothelial cells, diffuse perinuclear staining for Nox1 was observed (Sipkens et al., 2011), whereas in cultured vascular smooth muscle cells, Nox1 has been shown to be expressed at the plasma membrane, co-localized with caveolae (Hilenski et al., 2004). Furthermore, Nox1 is expressed in early endosomes in vascular smooth muscle cells (Miller et al., 2007b). Nox2 protein was found in lipid raft rich fractions from cultured endothelial cells (Zhang et al., 2006). Interestingly, in endothelial cells, there is both evidence for (Li and Shah, 2002) and against (Sipkens et al., 2011) perinuclear expression of Nox2. Finally, Nox4 has been identified in the nucleus (Hilenski et al., 2004; Kuroda et al., 2005) and the endoplasmic reticulum (Van Buul et al., 2005; Chen et al., 2008; Lyle et al., 2009) in vascular smooth muscle and endothelial cells. In addition, Nox4 has also been shown to be co-localized with focal adhesions in vascular smooth muscle (Hilenski et al., 2004; Lyle et al., 2009). While studies in cultured vascular cells are vital in our understanding of subcellular localization of the different isoforms of NADPH oxidase, it is important that these findings are confirmed in intact cerebral arteries.

## Effects of Ang II on cerebrovascular function

### Generation of reactive oxygen species

A well-known effect of exposure to Ang II in the vasculature is increased generation of ROS. Acute Ang II treatment increases ROS production by cerebral arteries from mice (Kazama et al., 2004; De Silva et al., 2009; Jackman et al., 2009) and rats (Miller et al., 2005). Furthermore, both Nox1 (Jackman et al., 2009) and Nox2 (Kazama et al., 2004; De Silva et al., 2009) contribute to Ang II stimulated ROS production as genetic deletion of the catalytic subunit of either of these isoforms reduces Ang II stimulated ROS production by cerebral arteries. In this regard, it is interesting to note that in non-cerebral arteries from Nox1-deficient mice, AT1 receptors fail to properly localize at the cell membrane (Basset et al., 2009), suggesting that Nox1-derived ROS may regulate expression of AT1 receptors at the cell surface. While it remains to be established if this same pattern occurs in cerebral vessels, it is conceivable that the attenuation in Ang II-stimulated O_2_^·−^ production found in cerebral arteries from Nox1-deficient mice may actually be due to impaired trafficking of the AT1 receptor to the cell membrane. The importance of Nox4 in generating ROS in response to Ang II in cerebral arteries is currently unknown. In addition to rapid effects *in vitro*, acute systemic administration of Ang II increases cerebrovascular ROS levels. Using confocal microscopy, acute Ang II infusion intravenously increased ROS production by cerebral blood vessels in the cerebral cortex that was dependent on activation of AT1 receptors and Nox2 NADPH oxidase (Girouard et al., 2006, 2007). Thus, acute *in vitro* or *in vivo* Ang II treatment increases cerebrovascular NADPH oxidase-derived ROS. The majority of evidence to date suggests Nox2 NADPH oxidase is the primary source of Ang II-stimulated ROS in cerebral arteries. However, there is some evidence to suggest that Ang II may also activate Nox1 NADPH oxidase in cerebral blood vessels.

In addition to acute stimulation of ROS production, Ang II elevates cerebrovascular ROS levels when administered more chronically. For example, cerebrovascular O_2_^·−^ production is elevated following 7-days Ang II treatment. Specifically, both basal and Nox2 stimulated O_2_^·−^ production by cerebral arteries was elevated following Ang II treatment (Chrissobolis et al., 2012). Consistent with previous studies that have reported a key role for Nox2 NADPH oxidase in Ang II-stimulated ROS production, ROS production by cerebral arteries from Nox2 deficient mice treated with Ang II was not elevated (Chrissobolis et al., 2012). In support of these findings, studies using genetically altered mice with life-long Ang II-dependent hypertension also suggest that increased O_2_^·−^ underlies impaired endothelial function in cerebral arteries (Faraci et al., 2006).

In another model of chronic Ang II-dependent hypertension, using a lower dose of Ang II to produce a delayed increase in blood pressure (also known as a “slow-pressor” dose of Ang II), ROS levels in the vasculature are elevated (Capone et al., 2010, 2011, 2012). Specifically, using dihydroethidium fluorescence (to detect ROS) and immunofluorescence (to stain for cell-specific markers), increases in ROS levels were localized to the endothelium as well as neurons. This elevation in ROS was associated with impaired cerebrovascular responses to endothelium-dependent agonists (Capone et al., 2011, 2012). Scavenging O_2_^·−^ reduced ROS levels and ameliorated the impaired vasodilation, indicating that the increases in O_2_^·−^ were functionally important (Capone et al., 2011). Using this same model, Ang II infusion produced an increase in oxidative stress within the subfornical organ (SFO) (Capone et al., 2012), a specialized circumventricular organ which can sense changes in plasma Ang II levels and participates in central mechanisms that promote hypertension. Increased oxidative stress in the SFO paralleled increases in circulating arginine vasopressin (AVP) and increased perivascular concentrations of endothelin-1 (ET-1) (Capone et al., 2012). Ultimately, both ET-1 and Ang II mediated the elevation in ROS in the vasculature (Capone et al., 2012), presumably via activation of Nox2 NADPH oxidase. Overall, this recent study suggested that the SFO, though effects on AVP and ET-1, contributes to Ang II-induced oxidative stress.

### Alterations to cerebrovascular function

In addition to being a strong stimulus of NADPH oxidase activity, Ang II is a potent vasoconstrictor. Studies of cerebral arteries, including those in humans, have found that Ang II produces vasoconstriction (Whalley et al., 1985, 1987; Toda et al., 1990; Bevan et al., 1998; Stenman and Edvinsson, 2004; Miller et al., 2005; Vincent et al., 2005; Faraci et al., 2006; De Silva et al., 2009; Amberg et al., 2010; Ahnstedt et al., 2011). Both genetic and pharmacological approaches have established that Ang II induces constriction of cerebral arteries via activation of AT1 receptors (Naveri et al., 1994; Stenman and Edvinsson, 2004; Faraci et al., 2006). Classical Ang II-induced contraction of smooth muscle occurs via AT1 receptor mediated activation of phospholipase C, resulting in inositol 1,4,5-triphosphate (IP_3_) and diacylglycerol (DAG) production. IP_3_ and DAG regulate two distinct pathways, however, both ultimately result in smooth muscle contraction via activation of a number of protein kinases, such as myosin light chain kinase and Rho-kinase (Hilgers and Webb, 2005). Indeed, Ang II-induced constriction of basilar arteries is abolished in the presence of the Rho-kinase inhibitor, Y-27632 (Faraci et al., 2006).

While some effects of Ang II can clearly be mediated by AT1 receptors on vascular muscle, double-label immunoelectron microscopy and other approaches have demonstrated that AT1 receptors are also present in cerebral endothelium where Ang II can produce endothelium-dependent constriction via cyclooxygenase- and/or O_2_^·−^-dependent mechanisms (Manabe et al., 1989; Kazama et al., 2004; De Silva et al., 2009). As noted above, ROS are important modulators of vascular tone and Ang II-induced constriction of cerebral arteries in mice are mediated by H_2_O_2_ (De Silva et al., 2009). Considering this and the aforementioned findings together, it is possible that Ang II-induced constriction of cerebral arteries occurs via stimulation of ROS production, which in turn leads to the activation of Rho-kinase. While Rho-kinase is not generally thought to be a redox sensitive enzyme, the activity of the small G-protein RhoA, the principal activator of Rho-kinase, is redox-sensitive and thus may be modulated by ROS. Indeed, both O_2_^·−^ and t-butyl hydroperoxide (a stable analog of H_2_O_2_) can activate RhoA (Jin et al., 2004; Jernigan et al., 2008; Aghajanian et al., 2009; Broughton et al., 2009). Specifically, oxidation of two cysteine residues in the phosphoryl binding loop of RhoA by *t*-butyl hydroperoxide results in the exchange of guanosine diphosphate (GDP) for guanosine triphosphate (GTP), and activation of RhoA (Aghajanian et al., 2009). Once active, RhoA then translocates to the cell membrane where it binds to and activates Rho-kinase (Leung et al., 1995). This may result in signaling via the well-established pathway of Rho-kinase-dependent inhibition of myosin light chain phosphatase (MLCP), thus preventing the de-phosphorylation of myosin (Kimura et al., 1996; Jin et al., 2004; Jernigan et al., 2008). While this may be a potential signaling pathway by which Ang II induced constriction in cerebral arteries, further studies are needed to clarify the role of ROS in vascular responses to Ang II.

**Figure 2:**
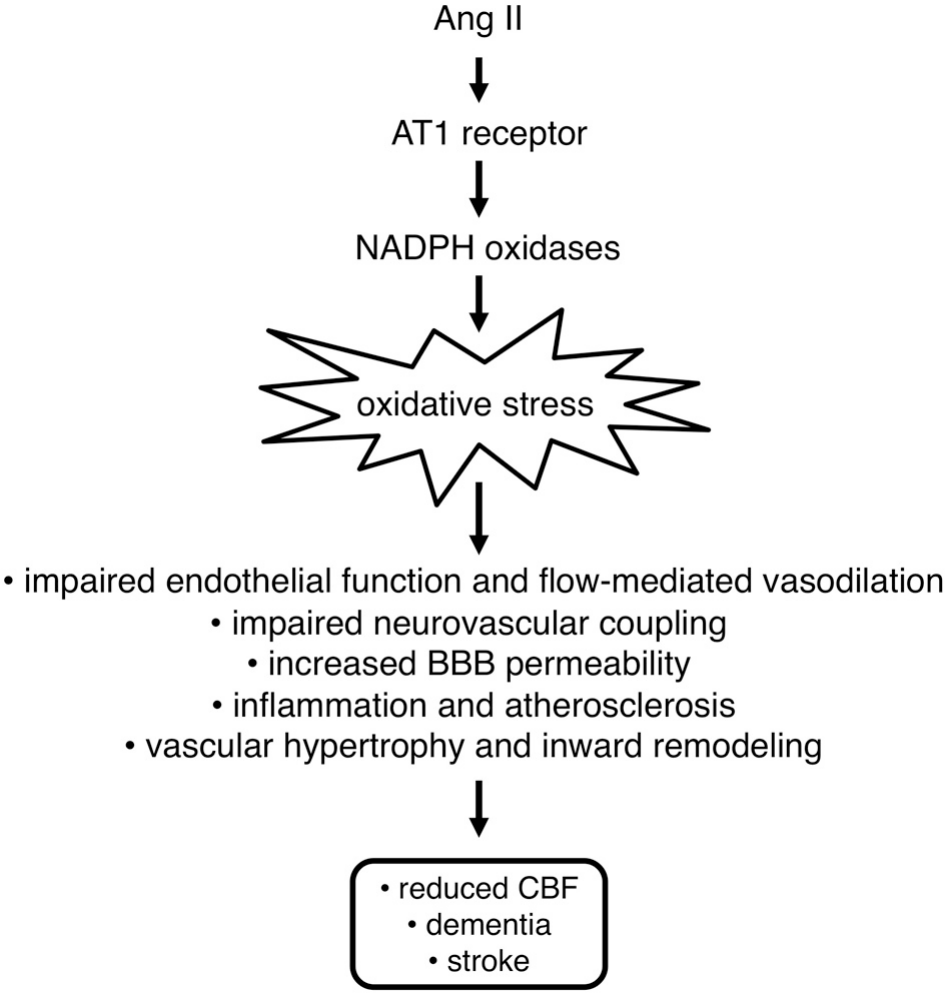
**Angiotensin II (Ang II) promotes oxidative stress in the vasculature via stimulation of AT1 receptors and subsequent activation of NADPH oxidases.** Oxidative stress contributes to blood-brain barrier (BBB) dysfunction, impairment of vasodilation and neurovascular coupling, and promotes inward vascular remodeling and inflammation. Overall, these effects contribute to reductions in cerebral blood flow, which can lead to dementia and increased susceptibility for stroke and lessen recovery following stroke or other forms of brain injury.

In addition to direct effects on vasomotor tone, Ang II impairs endothelium-dependent vasodilation by reducing the bioavailability of NO^·^ and potentially by interfering with other endothelium-dependent mechanisms. This effect is intimately linked with the increased production of ROS (mainly O_2_^·−^) in response to Ang II and resulting impairment of NO^·^-mediated signaling (Figure 2). Consistent with this concept, numerous studies indicate that Ang II has deleterious effects on cerebrovascular function. Indeed, in multiple models (both acute and chronic administration) (Gerzanich et al., 2003; Faraci et al., 2006; Kitayama et al., 2006; Chrissobolis and Faraci, 2010; Chrissobolis et al., 2012), Ang II impairs endothelium-dependent vasodilation cerebral blood vessels. Treatment with scavengers of O_2_^·−^ improved endothelial function, thus indicating that oxidative stress played a key role in producing this dysfunction (Faraci et al., 2006; Kitayama et al., 2006; Chrissobolis and Faraci, 2010). A recent study has provided, further evidence supporting a role for NADPH oxidase-derived ROS in causing oxidative stress in response to Ang II treatment. Specifically, 7-days Ang II treatment did not result in impaired endothelium-dependent vasodilatation in arteries from Nox2 deficient mice (Chrissobolis et al., 2012). This same study reported a small role for the Nox1-containing NADPH oxidase in this model of Ang II-induced dysfunction (Chrissobolis et al., 2012). Further studies will be needed to better define the role of this isoform of NADPH oxidase as well as Nox4- and Nox5-containing NADPH oxidase in hypertension as well as other disease states.

### Effects on neurovascular coupling

Many cell types including vascular muscle and endothelial cells, neurons and glia work together to supply oxygen and glucose to support the dynamic energy demands of the brain. This interaction between the cells within the parenchyma has given rise to the concept of a “neurovascular unit,” a multi-cellular structure with unique features and functions. For example, cells within the neurovascular unit are part of one segment of the circulation that contributes to regulation of cerebral blood flow during changes in metabolic demands that result from increased brain activity. This relationship between cellular activity and local cerebral blood flow is often termed neurovascular coupling or functional hyperemia. Cellular activity increases the need of brain cells for oxygen and nutrients, as well as the removal of metabolic by-products by the circulating blood. Specifically, when neurons and/or glia are active and require more energy substrates, the release of paracrine factors and other mechanisms produce dilation of local cerebral arterioles and upstream vessels supplying the region resulting in an increase in local cerebral blood flow. Several factors have been suggested to mediate the neurovascular coupling response. NO^·^ (Iadecola et al., 1995; Liu et al., 2008), K^+^ ions (Filosa et al., 2006), arachidonic acid metabolites (Niwa et al., 2000; Liu et al., 2008) as well as products of cellular metabolism such as adenosine (Dirnagl et al., 1994; Meno et al., 2001) have all been proposed to contribute to neurovascular coupling. These factors are release by neurons/glial cells and activate receptors/ion channels on cerebral blood vessels leading to vasodilation and a subsequent increase in local cerebral blood flow. It has also been suggested that the identity of the factor(s) mediating the neurovascular coupling response is dependent on the brain region and multiple agents may act in concert to produce the full vasodilatory response (Iadecola and Nedergaard, 2007).

Neurovascular coupling is affected by both Ang II and oxidative stress (Figure 2). Both acute and chronic systemic administration of Ang II increases arterial blood pressure. Irrespective of the length of treatment, Ang II-induced hypertension was associated with impairment of the neurovascular coupling response to whisker stimulation (Kazama et al., 2003, 2004; Girouard et al., 2007, 2008; Capone et al., 2009, 2010, 2011). Interestingly, in mice that were administered phenylephrine acutely to produce a similar degree of hypertension, there was no impairment of neurovascular coupling (Kazama et al., 2003; Capone et al., 2011), suggesting that Ang II and not the elevation in systemic pressure *per se* caused the neurovascular dysfunction. This impaired neurovascular coupling could be restored to normal by treatment with O_2_^·−^ scavengers (Kazama et al., 2004; Capone et al., 2011), suggesting a key role for oxidative stress. Further studies indicated that Nox2 NADPH oxidase was the likely source of increased ROS as Nox2-deficient mice were protected from Ang II-dependent neurovascular dysfunction (Kazama et al., 2004; Girouard et al., 2007). Ang II has also been shown to increase 3-nitrotyrosine immunoreactivity (a marker for ONOO^−^ formation), in a Nox2 NADPH oxidase-dependent manner in cerebral vessels (Girouard et al., 2007), suggesting that Ang II-dependent hypertension causes oxidative stress and vascular dysfunction within components of the neurovascular unit. Furthermore, it was also shown that a ONOO^−^ scavenger or a ONOO^−^ decomposition catalyst protect against Ang II dysfunction (Girouard et al., 2007). These findings suggest that ONOO^−^ may be a key intermediate of neurovascular dysfunction in response to Ang II.

The presence of the blood-brain barrier (BBB) is a unique characteristic of cerebral blood vessels with many functions including regulation of influx and efflux of cells and molecules between the blood and the brain. There is emerging evidence that Ang II and oxidative stress have detrimental effects on BBB permeability (Figure 2). For example, Ang II increases the permeability of cerebral microvascular endothelial cells in culture (Guillot and Audus, 1991; Fleegal-Demotta et al., 2009), which may contribute to increases in BBB permeability *in vivo*. Indeed, studies of Ang II-dependent hypertensive mice have found that BBB permeability is increased compared with vehicle treated mice (Vital et al., 2010; Zhang et al., 2010). Feeding of a high salt diet to Ang II treated mice did not augment BBB permeability, suggesting that Ang II and not increases in arterial pressure *per se* was the mediator of BBB dysfunction (Vital et al., 2010). Along these lines, DOCA (deoxycorticosterone acetate)/salt-induced hypertension is not associated with increased blood brain barrier permeability (Rodrigues and Granger, 2011). Oxidative stress is likely to be a key mediator of BBB dysfunction as treatment of Ang II-dependent hypertensive mice with a O_2_^·−^ scavenger prevents BBB dysfunction (Zhang et al., 2010). The source of ROS and the mechanisms by which BBB permeability is increased during Ang II-dependent hypertension remains unclear. However, considering that Nox1 and Nox2 NADPH oxidase are important sources of Ang II-stimulated ROS in the cerebral circulation, these enzymes may play a key role.

### Alterations to vascular structure

One of the mechanisms that can contribute to vascular dysfunction and impact cerebral blood flow is alterations to the structure of cerebral arteries and arterioles. Hypertension is associated with hypertrophy of large conduit vessels, such as the aorta, whereas it causes inward remodeling of smaller resistance vessels, including cerebral arterioles (Figure 2; Baumbach and Heistad, 1989; Baumbach et al., 2003). This latter structural change results in a smaller diameter lumen even when the vessel is maximally dilated (Baumbach and Heistad, 1989; Baumbach et al., 2003) and has emerged as a potential risk factor for cardiovascular and cerebrovascular events (Faraci, 2011). Ang II appears to be a critical mediator of inward remodeling as cerebral arterioles undergo remodeling and hypertrophy in Ang II-dependent models of hypertension, whereas Ang II-independent hypertension only causes hypertrophy (Baumbach et al., 2003). Furthermore, preliminary studies suggest Nox2 NADPH oxidase may be the critical link between Ang II and inward remodeling as cerebral arterioles from Nox2 deficient mice do not undergo inward remodeling following Ang II infusion (Chan and Baumbach, 2009). Inward remodeling impairs vasodilator capacity and thus can compromise brain perfusion and may contribute to impaired cognitive function in people with hypertension.

### Cerebrovascular inflammation

Hypertension induces an inflammatory phenotype within the cerebral vasculature and Ang II is believed to be a key mediator of this effect (Figure 2). For example, spontaneously hypertensive rats (SHR) have elevated expression of the pro-inflammatory marker intercellular adhesion molecule 1 (ICAM-1) (Ando et al., 2004). Interestingly, treatment of SHR with an AT1 receptor antagonist reduced the expression of ICAM-1, indicating a key role for Ang II (Ando et al., 2004). The increased expression of this adhesion molecule was associated with increased adhesion and infiltration of macrophages into the brain of SHR (Ando et al., 2004). As with ICAM-1 expression, treatment with an AT1 receptor antagonist prevented the increased macrophage infiltration (Ando et al., 2004). More recently, it has been demonstrated that there is an increase in leukocyte and platelet adhesion to the microvasculature in Ang II-induced hypertension (Vital et al., 2010; Zhang et al., 2010). The increased leukocyte/platelet adhesion observed during Ang II-induced hypertension is dependent on AT1 receptors on leukocytes/platelets (Vital et al., 2010) and ROS (Zhang et al., 2010), indicating that oxidative stress may play a key role. Similarly, in a DOCA/salt model of hypertension, leukocyte and platelet adhesion is dependent on activation of AT1 receptors and mitochondrial-derived ROS (Rodrigues and Granger, 2011). The source of the ROS contributing to augmented leukocyte and platelet adhesion to the cerebral microvasculature warrants further investigation. However, due to the link between AT1 receptors and Nox2 NADPH oxidase, it is possible that this isoform of NADPH oxidase plays a key role.

### Sex-dependent differences in cerebrovascular responses to Ang II

It is well-known that the incidence of cerebrovascular disease is lower in pre-menopausal females compared with males and post-menopausal females (Prencipe et al., 1997; Appelros et al., 2009). At present study, the precise reasons for the sex-dependent differences in disease incidence remain to be fully defined. However, the fact that Ang II has sexually dimorphic effects on the cerebral vasculature may contribute to the observed difference in disease incidence. Acute Ang II treatment increases O_2_^·−^ and H_2_O_2_ levels to a greater extent in arteries from males compared with females (De Silva et al., 2009). Indeed, sex differences in Ang II stimulated ROS was completely dependent on Nox2 NADPH oxidase (De Silva et al., 2009). This effect is consistent with other previous reports that have shown that Ang II exerts sexually dimorphic effects with regards to vasoconstriction (Faraci et al., 2006) and susceptibility to vascular dysfunction (Girouard et al., 2008; Chrissobolis and Faraci, 2010). This effect of sex on cerebrovascular ROS levels, vasoconstriction and neurovascular dysfunction in response to Ang II is likely due to the protective effects of estrogen on the cerebral circulation. Specifically, cerebrovascular ROS levels in response to Ang II are only elevated during diestrus (low estrogen levels) and Ang II does not significantly elevate ROS levels during proestrus and estrus (Capone et al., 2009). Due to the increase in oxidative stress, female mice in diestrus were susceptible to Ang II-induced neurovascular dysfunction (Capone et al., 2009). Currently, the mechanisms by which estrogen suppresses Ang II-induced oxidative stress in the cerebral circulation remains to be determined. If therapies are developed that can exploit this phenomenon, it may prove to be an effective therapy to reduce cerebrovascular oxidative stress.

### Effects on cerebral blood flow in humans

Hypertension has detrimental effects on cerebral blood flow in humans (Lipsitz et al., 2005; Beason-Held et al., 2007; Dai et al., 2008; Efimova et al., 2008; Nagata et al., 2010; Waldstein et al., 2010; Muller et al., 2012). Hypertension is associated with a shift in the autoregulatory curve to the right (Cipolla, 2007; Iadecola and Davisson, 2008). This means that during hypertension, higher pressures are needed to maintain cerebral perfusion in the normal range. Therefore, an aggressive blood pressure lowering regime may result in cerebral hypoperfusion and adverse outcomes. Despite the aforementioned concerns, a number of experimental studies have reported that anti-hypertensive therapies that target the RAS improve cerebral blood flow. As discussed above, Ang II has a number of diverse effects on the cerebral circulation, including effects on vascular tone, which may lead to reductions in cerebral blood flow (Figure 2). As such, it is not surprising that treatment with either ACE inhibitors (Lipsitz et al., 2005; Efimova et al., 2008) or AT1 receptor antagonists (Oku et al., 2005; Kimura et al., 2010; Nagata et al., 2010; Muller et al., 2012) improves or maintains total cerebral blood flow in hypertensive patients. Recently however, the Second Manifestations of ARTerial disease-Magnetic Resonance (SMART-MR) study reported that despite effective treatment (reduction of BP to <140/90 mmHg) neither ACE inhibitors or other non RAS based therapies (β-blocker, diuretic or calcium channel blocker) prevented the decline in cerebral blood flow that occurs in elderly hypertensive individuals over an average 3.9 years follow up period (Muller et al., 2012). In contrast, effective treatment (BP <140/90 mmHg) of hypertensive patients with an AT1 receptor antagonists prevented the decline in cerebral blood flow (Muller et al., 2012). The inability of ACE inhibitors to improve cerebral blood flow may suggest that AT1 receptor antagonism provides additional benefits over reducing circulating Ang II levels. One possibility is that inhibition of AT1 receptors allows circulating Ang II to signal via AT2 receptors. AT2 receptor activation has a number of potential beneficial effects on the vasculature, many of which oppose with effects of AT1 receptor activation (e.g., vasodilatation). As oxidative stress is a central component of Ang II-induced cerebrovascular dysfunction it would be expected that oxidative stress contributes to changes in cerebral blood flow during hypertension. However, further studies are needed to clarify whether oxidative stress plays a role in alterations to cerebral perfusion associated with hypertension in humans and which class of therapeutics would be of most benefit in reducing hypertension, while maintaining cerebral perfusion.

Increases in blood flow produce flow-mediated (or flow-dependent) dilation of cerebral arteries in rats *in vivo* (Fujii et al., 1991, 1992; Paravicini et al., 2006). A similar dilator mechanism appears to be present in humans and may be impacted by hypertension. For example, hypertension impairs increases in cerebral blood flow caused by mental stress (Naqvi and Hyuhn, 2009). Dilation of the common carotid artery as well as increases in blood flow velocity in the middle cerebral artery in response to mental stress are selectively impaired in hypertensive patients compared with normotensive controls (Naqvi and Hyuhn, 2009). These findings in humans are consistent with studies in animal models which suggest that hypertension impairs neurovascular coupling and mechanisms that provide critical support for functional hyperemia responses (flow-mediated dilation of large upstream vessels with the resulting maintenance of local microvascular pressure). If Ang II and oxidative stress similarly contribute to the impaired neurovascular coupling, therapeutically targeting the RAS may be of benefit.

## Conclusion

Ang II has prominent effects on the cerebral circulation. Many of these effects are deleterious and occur as a result of oxidative stress and the accompanying vascular dysfunction. Overall, the dysfunction caused by Ang II may result in compromised cerebral perfusion and may ultimately predispose the brain the hypoperfusion-dependent cognitive abnormalities as well as ischemic events. The vast majority of experimental studies to date implicate Nox2 NADPH oxidase-derived ROS as the major source of O_2_^·−^ and thus a key mediator of Ang II-induced oxidative stress. Due to the sensitivity of cerebral arteries to ROS, alterations to cerebrovascular function in response to Ang II may occur prior to changes in other parts of the vasculature. As such, additional studies that further define the role of Ang II and oxidative stress in the pathogenesis of cerebrovascular diseases will prove crucial. Furthermore, identification of molecules that protect the cerebral circulation and the brain against the effects of Ang II and oxidative stress may be of substantial benefit.

### Conflict of interest statement

The authors declare that the research was conducted in the absence of any commercial or financial relationships that could be construed as a potential conflict of interest.

## References

[B1] Aghajanian A. Wittchen E. S. Campbell S. L. Burridge K. (2009). Direct activation of RhoA by reactive oxygen species requires a redox-sensitive motif. PLoS ONE 4:e8045 10.1371/journal.pone.0008045 19956681 PMC2778012

[B2] Ago T. Kitazono T. Kurado J. Kumai Y. Kamouchi M. Ooboshi H. (2005). NAD(P)H oxidases in rat basilar arterial endothelial cells. Stroke 36, 1040–1046 10.1161/01.STR.0000163111.05825.0b 15845888

[B3] Ahnstedt H. Saveland H. Nilsson O. Edvinsson L. (2011). Human cerebrovascular contractile receptors are upregulated via a B-Raf/MEK/ERK-sensitive signaling pathway. BMC Neurosci. 12:5 10.1186/1471-2202-12-5 21223556 PMC3023719

[B4] Ambasta R. K. Kumar P. Griendling K. K. Schmidt H. H. Busse R. Brandes R. P. (2004). Direct interaction of the novel Nox proteins with p22phox is required for the formation of a functionally active NADPH oxidase. J. Biol. Chem. 279, 45935–45941 10.1074/jbc.M406486200 15322091

[B5] Amberg G. C. Earley S. Glapa S. A. (2010). Local regulation of arterial L-type calcium channels by reactive oxygen species. Circ. Res. 107, 1002–1010 10.1161/CIRCRESAHA.110.217018 20798361 PMC2967383

[B6] Ando H. Zhou J. Macova M. Imboden H. Saavedra J. M. (2004). Angiotensin II AT_1_ receptor blockade reverses pathological hypertrophy and inflammation in brain microvessels of spontaneously hypertensive rats. Stroke 35, 1726–1731 10.1161/01.STR.0000129788.26346.18 15143297

[B7] Appelros P. Stegmayr B. Terent A. (2009). Sex differences in stroke epidemiology: a systematic review. Stroke 40, 1082–1090 10.1161/STROKEAHA.108.540781 19211488

[B8] Banfi B. Clark R. Steger K. Krause K. (2003). Two novel proteins activate superoxide generation by the NADPH oxidase NOX1. J. Biol. Chem. 278, 3510–3513 10.1074/jbc.C200613200 12473664

[B9] Basset O. Deffert C. Foti M. Bedard K. Jaquet V. Ogier-Denis E. (2009). NADPH oxidase 1 deficiency alters caveolin phosphorylation and angiotensin II-receptor localization in vascular smooth muscle. Antioxid. Redox Signal. 11, 2371–2384 10.1089/ARS.2009.2584 19309260

[B10] Baumbach G. Heistad D. (1989). Remodeling of cerebral arterioles in chronic hypertension. Hypertension 13, 968–972 2737731 10.1161/01.hyp.13.6.968

[B11] Baumbach G. L. Sigmund C. D. Faraci F. M. (2003). Cerebral arteriolar structure in mice overexpressing human renin and angiotensinogen. Hypertension 41, 50–55 10.1161/01.HYP.0000042427.05390.5C 12511529

[B12] Beason-Held L. L. Moghekar A. Zonderman A. B. Kraut M. A. Resnick S. M. (2007). Longitudinal changes in cerebral blood flow in the older hypertensive brain. Stroke 38, 1766–1773 10.1161/STROKEAHA.106.477109 17510458

[B13] Bedard K. Krause K.-H. (2007). The NOX family of ROS-generating NADPH oxidases: physiology and pathophysiology. Physiol. Rev. 87, 245–313 10.1152/physrev.00044.2005 17237347

[B14] Bevan R. Dodge J. Nichols P. Poseno T. Vijayakumaran E. Wellman T. (1998). Responsiveness of human infant cerebral arteries to sympathetic nerve stimulation and vasoactive agents. Pediatr. Res. 44, 730–739 10.1203/00006450-199811000-00016 9803455

[B15] Broughton B. R. Jernigan N. L. Norton C. E. Walker B. R. Resta T. C. (2009). Chronic hypoxia augments depolarization-induced Ca^2+^ sensitization in pulmonary vascular smooth muscle through superoxide-dependent stimulation of RhoA. Am. J. Physiol. Lung Cell Mol. Physiol. 298, L232–L242 10.1152/ajplung.00276.2009 19897743 PMC2822557

[B16] Capone C. Anrather J. Milner T. A. Iadecola C. (2009). Estrous cycle-dependent neurovascular dysfunction induced by angiotensin II in the mouse neocortex. Hypertension 54, 302–307 10.1161/HYPERTENSIONAHA.109.133249 19506098 PMC2750855

[B17] Capone C. Faraco G. Anrather J. Zhou P. Iadecola C. (2010). Cyclooxygenase 1-derived prostaglandin E2 and EP1 receptors are required for the cerebrovascular dysfunction induced by angiotensin II. Hypertension 55, 911–917 10.1161/HYPERTENSIONAHA.109.145813 20194308 PMC2861995

[B18] Capone C. Faraco G. Park L. Cao X. Davisson R. L. Iadecola C. (2011). The cerebrovascular dysfunction induced by slow pressor doses of angiotensin-II precedes the development of hypertension. Am. J. Physiol. Heart Circ. Physiol. 300, H397–H407 10.1152/ajpheart.00679.2010 20971763 PMC3023263

[B19] Capone C. Faraco G. Peterson J. R. Coleman C. Anrather J. Milner T. A. (2012). Central cardiovascular circuits contribute to the neurovascular dysfunction in angiotensin II hypertension. J. Neurosci. 32, 4878–4886 10.1523/JNEUROSCI.6262-11.2012 22492044 PMC3328774

[B20] Chan S. Baumbach G. (2009). Nox2 deficiency prevents angiotensin II- induced hypertrophy and inward remodeling in cerebral arterioles (Abstract). FASEB J. 23, 11

[B21] Chen K. Kirber M. T. Xiao H. Yang Y. Keaney J. F. Jr. (2008). Regulation of ROS signal transduction by NADPH oxidase 4 localization. J. Cell Biol. 181, 1129–1139 10.1083/jcb.200709049 18573911 PMC2442210

[B22] Cheng G. Cao Z. Xu X. Meir E. G. V. Lambeth J. D. (2001). Homologs of gp91phox: cloning and tissue expression of Nox3, Nox4, and Nox5. Gene 269, 131–140 10.1016/S0378-1119(01)00449-8 11376945

[B23] Choi H. Leto T. L. Hunyady L. Catt K. J. Bae Y. S. Rhee S. G. (2008). Mechanism of angiotensin II-induced superoxide production in cells reconstituted with angiotensin type 1 receptor and the components of NADPH oxidase. J. Biol. Chem. 283, 255–267 10.1074/jbc.M708000200 17981802

[B24] Chrissobolis S. Banfi B. Sobey C. G. Faraci F. M. (2012). Role of Nox isoforms in angiotensin II-induced oxidative stress and endothelial dysfunction in brain. J. Appl. Physiol. 113, 184–191 10.1152/japplphysiol.00455.2012 22628375 PMC3774474

[B25] Chrissobolis S. Faraci F. M. (2008). The role of oxidative stress and NADPH oxidase in cerebrovascular disease. Trends Mol. Med. 14, 495–502 10.1016/j.molmed.2008.09.003 18929509 PMC3140460

[B26] Chrissobolis S. Faraci F. M. (2010). Sex differences in protection against angiotensin II-induced endothelial dysfunction by manganese superoxide dismutase in the cerebral circulation. Hypertension 55, 905–910 10.1161/HYPERTENSIONAHA.109.147041 20194298 PMC2866174

[B27] Cipolla M. J. (2007). Cerebrovascular function in pregnancy and eclampsia. Hypertension 50, 14–24 10.1161/HYPERTENSIONAHA.106.079442 17548723

[B28] Dai W. Lopez O. L. Carmichael O. T. Becker J. T. Kuller L. H. Gach H. M. (2008). Abnormal regional cerebral blood flow in cognitively normal elderly subjects with hypertension. Stroke 39, 349–354 10.1161/STROKEAHA.107.495457 18174483 PMC2701215

[B29] Deleo F. R. Yu L. Burritt J. B. Loetterle L. R. Bond C. W. Jesaitis A. J. (1995). Mapping sites of interaction of p47-phox and flavocytochrome b with random-sequence peptide phage display libraries. Proc. Natl. Acad. Sci. U.S.A. 92, 7110–7114 7624379 10.1073/pnas.92.15.7110 PMC41481

[B30] De Silva T. M. Broughton B. R. S. Drummond G. R. Sobey C. G. Miller A. A. (2009). Gender influences cerebral vascular responses to angiotensin II through Nox2-derived reactive oxygen species. Stroke 40, 1091–1097 10.1161/STROKEAHA.108.531707 19211495

[B31] Didion S. Faraci F. (2002). Effects of NADH and NADPH on superoxide levels and cerebral vascular tone. Am. J. Physiol. Heart Circ. Physiol. 282, H688–H695 10.1152/ajpheart.00576.2001 11788419

[B32] Didion S. Hathaway C. Faraci F. (2001). Superoxide levels and function of cerebral blood vessels after inhibition of CuZn-SOD. Am. J. Physiol. Heart Circ. Physiol. 281, H1697–H1703 11557560 10.1152/ajpheart.2001.281.4.H1697

[B33] Dikalova A. E. Gongora M. C. Harrison D. G. Lambeth J. D. Dikalov S. Griendling K. K. (2010). Upregulation of Nox1 in vascular smooth muscle leads to impaired endothelium-dependent relaxation via eNOS uncoupling. Am. J. Physiol. Heart Circ. Physiol. 299, H673–H679 10.1152/ajpheart.00242.2010 20639222 PMC2944492

[B34] Dirnagl U. Niwa K. Lindauer U. Villringer A. (1994). Coupling of cerebral blood flow to neuronal activation: role of adenosine and nitric oxide. Am. J. Physiol. Heart Circ. Physiol. 267, H296–H301 8048594 10.1152/ajpheart.1994.267.1.H296

[B35] Drummond G. R. Selemidis S. Griendling K. K. Sobey C. G. (2011). Combating oxidative stress in vascular disease: NADPH oxidases as therapeutic targets. Nat. Rev. Drug Discov. 10, 453–471 10.1038/nrd3403 21629295 PMC3361719

[B36] Efimova I. Y. Efimova N. Y. Triss S. V. Lishmanov Y. B. (2008). Brain perfusion and cognitive function changes in hypertensive patients. Hypertens. Res. 31, 673–678 10.1291/hypres.31.673 18633179

[B37] Fan L. M. Teng L. Li J.-M. (2009). Knockout of p47phox uncovers a critical role of p40phox in reactive oxygen species production in microvascular endothelial cells. Arterioscler. Thromb. Vasc. Biol. 29, 1651–1656 10.1161/ATVBAHA.109.191502 19608974 PMC2888064

[B38] Faraci F. M. (2006). Hydrogen peroxide: watery fuel for change in vascular biology. Arterioscler. Thromb. Vasc. Biol. 26, 1931–1933 10.1161/01.ATV.0000238355.56172.b3 16917110

[B39] Faraci F. M. (2011). Protecting against vascular disease in brain. Am. J. Physiol. Heart Circ. Physiol. 300, H1566–H1582 10.1152/ajpheart.01310.2010 21335467 PMC3094081

[B40] Faraci F. M. Lamping K. G. Modrick M. L. Ryan M. J. Sigmund C. D. Didion S. P. (2006). Cerebral vascular effects of angiotensin II: new insights from genetic models. J. Cereb. Blood Flow Metab. 26, 449–455 10.1038/sj.jcbfm.9600204 16094317

[B41] Filosa J. A. Bonev A. D. Straub S. V. Meredith A. L. Wilkerson M. K. Aldrich R. W. (2006). Local potassium signaling couples neuronal activity to vasodilation in the brain. Nat. Neurosci. 9, 1397–1403 10.1038/nn1779 17013381

[B42] Fleegal-Demotta M. A. Doghu S. Banks W. A. (2009). Angiotensin II modulates BBB permeability via activation of the AT1 receptor in brain endothelial cells. J. Cereb. Blood Flow Metab. 29, 640–647 10.1038/jcbfm.2008.158 19127280

[B43] Fontayne A. Dang P. M. Gougerot-Pocidalo M. A. El-Benna J. (2002). Phosphorylation of p47phox sites by PKC alpha, beta II, delta, and zeta: effect on binding to p22phox and on NADPH oxidase activation. Biochemistry 41, 7743–7750 10.1021/bi011953s 12056906

[B44] Fujii K. Heistad D. D. Faraci F. M. (1991). Flow-mediated dilatation of the basilar artery *in vivo*. Circ. Res. 69, 697–705 10.1161/01.RES.69.3.697 1873864

[B45] Fujii K. Heistad D. D. Faraci F. M. (1992). Effect of diabetes mellitus on flow-mediated and endothelium-dependent dilatation of the rat basilar artery. Stroke 23, 1494–1498 10.1161/01.STR.23.10.1494 1412587

[B46] Geiszt M. S. Kopp J. B. Varnai P. T. Leto T. L. (2000). Identification of Renox, an NAD(P)H oxidase in kidney. Proc. Natl. Acad. Sci. U.S.A. 97, 8010–8014 10.1073/pnas.130135897 10869423 PMC16661

[B47] Gerzanich V. Ivanova S. Zhou H. Simard J. M. (2003). Mislocalization of eNOS and upregulation of cerebral vascular Ca^2+^ channel activity in angiotensin-hypertension. Hypertension 41, 1124–1130 10.1161/01.HYP.0000066288.20169.21 12668586

[B48] Girouard H. Lessard A. Capone C. Milner T. A. Iadecola C. (2008). The neurovascular dysfunction induced by angiotensin II in the mouse neocortex is sexually dimorphic. Am. J. Physiol. Heart Circ. Physiol. 294, H156–H163 10.1152/ajpheart.01137.2007 17982007

[B49] Girouard H. Park L. Anrather J. Zhou P. Iadecola C. (2006). Angiotensin II attenuates endothelium-dependent responses in the cerebral microcirculation through Nox-2-derived radicals. Arterioscler. Thromb. Vasc. Biol. 26, 826–832 10.1161/01.ATV.0000205849.22807.6e 16439707

[B50] Girouard H. Park L. Anrather J. Zhou P. Iadecola C. (2007). Cerebrovascular nitrosative stress mediates neurovascular and endothelial dysfunction induced by angiotensin II. Arterioscler. Thromb. Vasc. Biol. 27, 303–309 10.1161/01.ATV.0000253885.41509.25 17138940

[B51] Groemping Y. Lapouge K. Smerdon S. J. Rittinger K. (2003). Molecular basis of phosphorylation-induced activation of the NADPH oxidase. Cell 113, 343–355 10.1016/S0092-8674(03)00314-3 12732142

[B52] Guillot F. L. Audus K. L. (1991). Angiotensin peptide regulation of bovine brain microvessel endothelial cell monolayer permeability. J. Cardiovasc. Pharmacol. 18, 212–218 1717781 10.1097/00005344-199108000-00006

[B53] Hilenski L. L. Clempus R. E. Quinn M. T. Lambeth J. D. Griendling K. K. (2004). Distinct subcellular localizations of Nox1 and Nox4 in vascular smooth muscle cells. Arterioscler. Thromb. Vasc. Biol. 24, 677–683 10.1161/01.ATV.0000112024.13727.2c 14670934

[B54] Hilgers R. H. P. Webb R. C. (2005). Molecular aspects of arterial smooth muscle contraction: focus on rho. Exp. Biol. Med. 230, 829–835 16339747 10.1177/153537020523001107

[B55] Iadecola C. Davisson R. L. (2008). Hypertension and cerebrovascular dysfunction. Cell Metab. 7, 476–484 10.1016/j.cmet.2008.03.010 18522829 PMC2475602

[B56] Iadecola C. Nedergaard M. (2007). Glial regulation of the cerebral microvasculature. Nat. Neurosci. 10, 1369–1376 10.1038/nn2003 17965657

[B57] Iadecola C. Li J. Ebner T. J. Xu X. (1995). Nitric oxide contributes to functional hyperemia in cerebellar cortex. Am. J. Physiol. Regul. Integr. Comp. Physiol. 268, R1153–R1162 7539595 10.1152/ajpregu.1995.268.5.R1153

[B58] Jackman K. A. Miller A. A. Drummond G. R. Sobey C. G. (2009). Importance of NOX1 for angiotensin II-induced cerebrovascular superoxide production and cortical infarct volume following ischemic stroke. Brain Res. 1286, 215–220 10.1016/j.brainres.2009.06.056 19559686

[B59] Jernigan N. L. Walker B. R. Resta T. C. (2008). Reactive oxygen species mediate RhoA/Rho kinase-induced Ca^2+^ sensitization in pulmonary vascular smooth muscle following chronic hypoxia. Am. J. Physiol. Lung Cell Mol. Physiol. 295, L515–L529 10.1152/ajplung.00355.2007 18621909 PMC2536792

[B60] Jin L. Ying Z. Webb R. C. (2004). Activation of Rho/Rho kinase signaling pathway by reactive oxygen species in rat aorta. Am. J. Physiol. Heart Circ. Physiol. 287, H1495–H1500 10.1152/ajpheart.01006.2003 15371261

[B61] Kawahara T. Ritsick D. Cheng G. Lambeth J. D. (2005). Point mutations in the proline-rich region of p22phox are dominant inhibitors of Nox1- and Nox2-dependent reactive oxygen generation. J. Biol. Chem. 280, 31859–31869 10.1074/jbc.M501882200 15994299

[B62] Kazama K. Anrather J. Zhou P. Girouard H. Frys K. Milner T. A. (2004). Angiotensin II impairs neurovascular coupling in neocortex through NADPH oxidase-derived radicals. Circ. Res. 95, 1019–1026 10.1161/01.RES.0000148637.85595.c5 15499027

[B63] Kazama K. Wang G. Frys K. Anrather J. Iadecola C. (2003). Angiotensin II attenuates functional hyperemia in the mouse somatosensory cortex. Am. J. Physiol. Heart Circ. Physiol. 285, H1890–H1899 10.1152/ajpheart.00464.2003 12907423

[B64] Kimura K. Ito M. Amano M. Chihara K. Fukata Y. Nakafuku M. (1996). Regulation of myosin phosphatase by Rho and Rho-associated kinase (Rho-kinase). Science 273, 245–248 10.1126/science.273.5272.245 8662509

[B65] Kimura Y. Kitagawa K. Oku N. Kajimoto K. Kato H. Tanaka M. (2010). Blood pressure lowering with valsartan is associated with maintenance of cerebral blood flow and cerebral perfusion reserve in hypertensive patients with cerebral small vessel disease. J. Stroke Cerebrovasc. Dis. 19, 85–91 10.1016/j.jstrokecerebrovasdis.2009.03.010 20189083

[B66] Kinugawa S. Huang H. Wang Z. Kaminski P. M. Wolin M. S. Hintze T. H. (2005). A defect of neuronal nitric oxide synthase increases xanthine oxidase-derived superoxide anion and attenuates the control of myocardial oxygen consumption by nitric oxide derived from endothelial nitric oxide synthase. Circ. Res. 96, 355–362 10.1161/01.RES.0000155331.09458.A7 15637297

[B67] Kitayama J. Faraci F. M. Lentz S. R. Heistad D. D. (2007). Cerebral vascular dysfunction during hypercholesterolemia. Stroke 38, 2136–2141 10.1161/STROKEAHA.107.481879 17525390

[B68] Kitayama J. Yi C. Faraci F. M. Heistad D. D. (2006). Modulation of dilator responses of cerebral arterioles by extracellular superoxide dismutase. Stroke 37, 2802–2806 10.1161/01.STR.0000245134.12145.ae 17008608

[B69] Kivipelto M. Helkala E. L. Laakso M. P. Hanninen T. Hallikainen M. Alhainen K. (2001). Midlife vascular risk factors and Alzheimer's disease in later life: longitudinal, population based study. BMJ 322, 1447–1451 10.1136/bmj.322.7300.1447 11408299 PMC32306

[B70] Kleinschnitz C. Grund H. Wingler K. Armitage M. E. Jones E. Mittal M. (2010). Post-stroke inhibition of induced NADPH oxidase type 4 prevents oxidative stress and neurodegeneration. PLoS Biol. 8:e1000479 10.1371/journal.pbio.1000479 20877715 PMC2943442

[B71] Kuroda J. Nakagawa K. Yamasaki T. Nakamura K.-I. Takeya R. Kuribayashi F. (2005). The superoxide-producing NAD(P)H oxidase Nox4 in the nucleus of human vascular endothelial cells. Genes Cell 10, 1139–1151 10.1111/j.1365-2443.2005.00907.x 16324151

[B72] Landmesser U. Dikalov S. Price S. R. Mccann L. Fukai T. Holland S. M. (2003). Oxidation of tetrahydrobiopterin leads to uncoupling of endothelial cell nitric oxide synthase in hypertension. J. Clin. Invest. 111, 1201–1209 10.1172/JCI14172 12697739 PMC152929

[B73] Leung T. Manser E. Tan L. Lim L. (1995). A novel serine/threonine kinase binding the Ras-related RhoA GTPase which translocates the kinase to peripheral membranes. J. Biol. Chem. 270, 29051–29054 10.1074/jbc.270.49.29051 7493923

[B74] Lewington S. Clarke R. Qizilbash N. Peto R. Collins R. Collaboration P. S. (2002). Age-specific relevance of usual blood pressure to vascular mortality: a meta-analysis of individual data for one million adults in 61 prospective studies. Lancet 360, 1903–1913 10.1016/S0140-6736(02)11911-8 12493255

[B75] Li J. M. Shah A. M. (2002). Intracellular localization and preassembly of the NADPH oxidase complex in cultured endothelial cells. J. Biol. Chem. 277, 19952–19960 10.1074/jbc.M110073200 11893732

[B76] Lipsitz L. A. Gagnon M. Vyas M. Iloputaife I. Kiely D. K. Sorond F. (2005). Antihypertensive therapy increases cerebral blood flow and carotid distensibility in hypertensive elderly subjects. Hypertension 45, 216–221 10.1161/01.HYP.0000153094.09615.11 15655124

[B77] Liu X. Li C. Falck J. R. Roman R. J. Harder D. R. Koehler R. C. (2008). Interaction of nitric oxide, 20-HETE, and EETs during functional hyperemia in whisker barrel cortex. Am. J. Physiol. Heart Circ. Physiol. 295, H619–H631 10.1152/ajpheart.01211.2007 18502903 PMC2519225

[B78] Lyle A. N. Deshpande N. N. Taniyama Y. Seidel-Rogol B. Pounkova L. Du P. (2009). Poldip2, a novel regulator of Nox4 and cytoskeletal integrity in vascular smooth muscle cells. Cir. Res. 105, 249–259 10.1161/CIRCRESAHA.109.193722 19574552 PMC2744198

[B79] Manabe K. Shirahase H. Usui H. Kurahashi K. Fujiwara M. (1989). Endothelium-dependent contractions induced by angiotensin I and angiotensin II in canine cerebral artery. J. Pharmacol. Exp. Ther. 251, 317–320 2795464

[B80] Martyn K. Frederick L. Von Loehneysen K. Dinauer M. Knaus U. (2006). Functional analysis of Nox4 reveals unique characteristics compared to other NADPH oxidases. Cell. Signal. 18, 69–82 10.1016/j.cellsig.2005.03.023 15927447

[B81] Meno J. R. Crum A. V. Winn H. R. (2001). Effect of adenosine receptor blockade on pial arteriolar dilation during sciatic nerve stimulation. Am. J. Physiol. Heart Circ. Physiol. 281, H2018–H2027 11668063 10.1152/ajpheart.2001.281.5.H2018

[B82] Miller A. A. Budzyn K. Sobey C. G. (2010). Vascular dysfunction in cerebrovascular disease: mechanisms and therapeutic intervention. Clin. Sci. (Lond.) 119, 1–17 10.1042/CS20090649 20370718

[B83] Miller A. A. Drummond G. R. De Silva T. M. Mast A. E. Hickey H. Williams J. P. (2009). NADPH oxidase activity is higher in cerebral versus systemic arteries of four animal species: role of Nox2. Am. J. Physiol. Heart Circ. Physiol. 296, H220–H225 10.1152/ajpheart.00987.2008 19028794

[B84] Miller A. A. Drummond G. R. Sobey C. G. (2006). Novel isoforms of NADPH-oxidase in cerebral vascular control. Pharmacol. Ther. 111, 928–948 10.1016/j.pharmthera.2006.02.005 16616784

[B85] Miller A. A. Drummond G. R. Mast A. E. Schmidt H. H. H. W. Sobey C. G. (2007a). Effect of gender on NADPH-oxidase activity, expression, and function in the cerebral circulation: role of estrogen. Stroke 38, 2142–2149 10.1161/STROKEAHA.106.477406 17525399

[B86] Miller F. J. Filali M. Huss G. J. Stanic B. Chamseddine A. Barna T. J. (2007b). Cytokine activation of nuclear factor κ B in vascular smooth muscle cells requires signaling endosomes containing Nox1 and ClC-3. Circ. Res. 101, 663–671 10.1161/CIRCRESAHA.107.151076 17673675

[B87] Miller A. Drummond G. Schmidt H. Sobey C. (2005). NADPH oxidase activity and function are profoundly greater in cerebral versus systemic arteries. Circ. Res. 97, 1055–1062 10.1161/01.RES.0000189301.10217.87 16210546

[B88] Modrick M. L. Didion S. P. Lynch C. M. Dayal S. Lentz S. R. Faraci F. M. (2009). Role of hydrogen peroxide and the impact of glutathione peroxidase-1 in regulation of cerebral vascular tone. J. Cereb. Blood Flow Metab. 29, 1130–1137 10.1038/jcbfm.2009.37 19352401 PMC2852621

[B89] Muller M. Van Der Graaf Y. Visseren F. L. Mali W. P. T. M. Geerlings M. I. For The Smart Study Group (2012). Hypertension and longitudinal changes in cerebral blood flow: the SMART-MR study. Ann. Neurol. 71, 825–833 10.1002/ana.23554 22447734

[B90] Nagata R. Kawabe K. Ikeda K. (2010). Olmesartan, an angiotensin II receptor blocker, restores cerebral hypoperfusion in elderly patients with hypertension. J. Stroke Cerebrovasc. Dis. 19, 236–240 10.1016/j.jstrokecerebrovasdis.2009.08.004 20434053

[B91] Naqvi T. Z. Hyuhn H. K. (2009). Cerebrovascular mental stress reactivity is impaired in hypertension. Cardiovasc. Ultrasound 7:32 10.1186/1476-7120-7-32 19575779 PMC2710316

[B92] Narayanan D. Xi Q. Pfeffer L. M. Jaggar J. H. (2010). Mitochondria control functional CaV1.2 expression in smooth muscle cells of cerebral arteries. Circ. Res. 107, 631–641 10.1161/CIRCRESAHA.110.224345 20616314 PMC3050675

[B93] Naveri L. Stromberg C. Saavedra J. M. (1994). Angiotensin II AT1 receptor mediated contraction of the perfused rat cerebral artery. Neuroreport 5, 2278–2280 7881045 10.1097/00001756-199411000-00018

[B94] Nisimoto Y. Motalebi S. Han C.-H. Lambeth J. D. (1999). The p67 phox activation domain regulates electron flow from NADPH to flavin in flavocytochrome b558. J. Biol. Chem. 274, 22999–23005 10.1074/jbc.274.33.22999 10438466

[B95] Niwa K. Araki E. Morham S. G. Ross M. E. Iadecola C. (2000). Cyclooxygenase-2 contributes to functional hyperemia in whisker-barrel cortex. J. Neurosci. 20, 763–770 10632605 10.1523/JNEUROSCI.20-02-00763.2000 PMC6772412

[B96] Niwa K. Haensel C. Ross M. E. Iadecola C. (2001). Cyclooxygenase-1 participates in selected vasodilator responses of the cerebral circulation. Circ. Res. 88, 600–608 10.1161/01.RES.88.6.600 11282894

[B97] Nobuhisa I. Takeya R. Ogura K. Ueno N. Kohda D. Inagaki F. (2006). Activation of the superoxide-producing phagocyte NADPH oxidase requires co-operation between the tandem SH3 domains of p47phox in recognition of a polyproline type II helix and an adjacent alpha-helix of p22phox. Biochem. J. 396, 183–192 10.1042/BJ20051899 16460309 PMC1449995

[B98] O'Donnell M. J. Xavier D. Liu L. Zhang H. Chin S. L. Rao-Melacini P. (2010). Risk factors for ischaemic and intracerebral haemorrhagic stroke in 22 countries (the INTERSTROKE Study): a case-control study. Lancet 376, 112–123 10.1016/S0140-6736(10)60834-3 20561675

[B99] Oku N. Kitagawa K. Imaizumi M. Takasawa M. Piao R. Kimura Y. (2005). Hemodynamic influences of losartan on the brain in hypertensive patients. Hypertens. Res. 28, 43–49 10.1291/hypres.28.43 15969254

[B100] Paravicini T. Chrissobolis S. Drummond G. Sobey C. (2004). Increased NADPH-oxidase activity and Nox4 expression during chronic hypertension is associated with enhanced cerebral vasodilatation to NADPH *in vivo*. Stroke 35, 584–589 10.1161/01.STR.0000112974.37028.58 14739416

[B101] Paravicini T. Miller A. Drummond G. Sobey C. (2006). Flow-induced cerebral vasodilatation *in vivo* involves activation of phosphatidylinositol-3 kinase, NADPH-oxidase, and nitric oxide synthase. J. Cereb. Blood Flow Metab. 26, 836–845 10.1038/sj.jcbfm.9600235 16222243

[B102] Park L. Anrather J. Zhou P. Frys K. Wang G. Iadecola C. (2004). Exogenous NADPH increases cerebral blood flow through NADPH oxidase-dependent and -independent mechanisms. Arterioscler. Thromb. Vasc. Biol. 24, 1860–1865 10.1161/01.ATV.0000142446.75898.44 15308559

[B103] Prencipe M. Ferretti C. Casini A. Santini M. Giubilei F. Culasso F. (1997). Stroke, disability and dementia: results of a population survey. Stroke 28, 531–536 10.1161/01.STR.28.3.531 9056607

[B104] Rodrigues S. F. Granger D. N. (2011). Cerebral microvascular inflammation in DOCA salt-induced hypertension: role of angiotensin II and mitochondrial superoxide. J. Cereb. Blood Flow Metab. 32, 368–375 10.1038/jcbfm.2011.139 21971354 PMC3272604

[B105] Roger V. L. Go A. S. Lloyd-Jones D. M. Adams R. J. Berry J. D. Brown T. M. (2011). Heart disease and stroke statistics—2011 update. Circulation 123, e18–e209 10.1161/CIR.0b013e3182009701 21160056 PMC4418670

[B106] Santhanam A. V. R. D'Uscio L. V. Smith L. A. Katusic Z. S. (2012). Uncoupling of eNOS causes superoxide anion production and impairs NO signaling in the cerebral microvessels of hph-1 mice. J. Neurochem. 122, 1211–1218 10.1111/j.1471-4159.2012.07872.x 22784235 PMC3433644

[B107] Sarfstein R. Gorzalczany Y. Mizrahi A. Berdichevsky Y. Molshanski-Mor S. Weinbaum C. (2004). Dual role of rac in the assembly of NADPH oxidase, tethering to the membrane and activation of p67phox. J. Biol. Chem. 279, 16007–16016 10.1074/jbc.M312394200 14761978

[B108] Schröder K. Zhang M. Benkhoff S. Mieth A. Pliquett R. Kosowski J. (2012). Nox4 Is a protective reactive oxygen species generating vascular NADPH oxidase. Circ. Res. 110, 1217–1225 10.1161/CIRCRESAHA.112.267054 22456182

[B109] Selemidis S. Sobey C. G. Wingler K. Schmidt H. H. Drummond G. R. (2008). NADPH oxidases in the vasculature: molecular features, roles in disease and pharmacological inhibition. Pharmacol. Ther. 120, 254–291 10.1016/j.pharmthera.2008.08.005 18804121

[B110] Sipkens J. A. Hahn N. Van Den Brand C. S. Meischl C. Cillessen S. A. Smith D. E. (2011). Homocysteine-Induced Apoptosis in Endothelial Cells Coincides With Nuclear NOX2 and Peri-nuclear NOX4 Activity. Cell Biochem. Biophys. [Epub ahead of print]. 10.1007/s12013-011-9297-y 22038300 PMC3825580

[B111] Stenman E. Edvinsson L. (2004). Cerebral ischemia enhances vascular angiotensin AT1 receptor-mediated contraction in rats. Stroke 35, 970–974 10.1161/01.STR.0000121642.53822.58 15001791

[B112] Taylor W. R. Jones D. T. Segal A. W. (1993). A structural model for the nucleotide binding domains of the flavocytochrome b-245 beta-chain. Protein Sci. 2, 1675–1685 10.1002/pro.5560021013 8251942 PMC2142254

[B113] Thomson L. Trujillo M. Telleri R. Radi R. (1995). Kinetics of cytochrome c2+ oxidation by peroxynitrite: implications for superoxide measurements in nitric oxide-producing biological systems. Arch. Biochem. Biophys. 319, 491–497 10.1006/abbi.1995.1321 7786032

[B114] Toda N. Ayaziki K. Okamura T. (1990). Modifications by endogenous prostaglandins of angiotensin II-induced contractions in dog and monkey cerebral and mesenteric arteries. J. Pharmacol. Exp. Ther. 252, 374–379 2299599

[B115] Van Buul J. D. Fernandez-Borja M. Anthony E. C. Hordijk P. L. (2005). Expression and localization of NOX2 and NOX4 in primary human endothelial cells. Antioxid. Redox Signal. 7, 308–317 10.1089/ars.2005.7.308 15706079

[B116] Vasquez-Vivar J. Kalyanaraman B. Martã¡Sek P. Hogg N. Masters B. S. S. Karoui H. (1998). Superoxide generation by endothelial nitric oxide synthase: the influence of cofactors. Proc. Natl. Acad. Sci. U.S.A. 95, 9220–9225 9689061 10.1073/pnas.95.16.9220 PMC21319

[B117] Vasquez-Vivar J. Martasek P. Hogg N. Masters B. S. Pritchard K. A. Jr. Kalyanaraman B. (1997). Endothelial nitric oxide synthase-dependent superoxide generation from adriamycin. Biochemistry 36, 11293–11297 10.1021/bi971475e 9333325

[B118] Vincent J.-M. Kwan Y. W. Lung Chan S. Perrin-Sarrado C. Atkinson J. Chillon J.-M. (2005). Constrictor and dilator effects of angiotensin II on cerebral arterioles. Stroke 36, 2691–2695 10.1161/01.STR.0000190002.79052.bf 16269635

[B119] Vital S. A. Terao S. Nagai M. Granger D. N. (2010). Mechanisms underlying the cerebral microvascular responses to angiotensin II-induced hypertension. Microcirculation 17, 641–649 10.1111/j.1549-8719.2010.00060.x 21044218 PMC3058857

[B120] Waldstein S. R. Lefkowitz D. M. Siegel E. L. Rosenberger W. F. Spencer R. J. Tankard C. F. (2010). Reduced cerebral blood flow in older men with higher levels of blood pressure. J. Hypertens. 28, 993–998 10.1097/HJH.0b013e328335c34f 20408259 PMC2878614

[B121] Whalley E. T. Amure Y. O. Lye R. H. (1987). Analysis of the mechanism of action of bradykinin on human basilar artery *in vitro*. Naunyn Schmiedebergs Arch. Pharmacol. 335, 433–437 3037391 10.1007/BF00165559

[B122] Whalley E. T. Paul K. S. Gulati O. P. (1985). Anti-spasmogenic effects of bencianol (ZY15051) on human cerebral arteries *in vitro*. Cephalalgia 5, 217–221 10.1046/j.1468-2982.1985.0504217.x 4084977

[B123] Zhang A. Y. Yi F. Zhang G. Gulbins E. Li P.-L. (2006). Lipid raft clustering and redox signaling platform formation in coronary arterial endothelial cells. Hypertension 47, 74–80 10.1161/10.1161/01.HYP.0000196727.53300.62 16344372

[B124] Zhang M. Mao Y. Ramirez S. H. Tuma R. F. Chabrashvili T. (2010). Angiotensin II induced cerebral microvascular inflammation and increased blood–brain barrier permeability via oxidative stress. Neuroscience 171, 852–858 10.1016/j.neuroscience.2010.09.029 20870012

